# Low FXIII activity levels in intensive care unit hospitalized COVID-19 patients

**DOI:** 10.1186/s12959-021-00333-3

**Published:** 2021-11-04

**Authors:** Yael Lichter, Tanya Badelbayov, Irina Shalev, Reut Schvartz, Yishay Szekely, Dan Benisty, Ilana Goldiner, Maxim Kagarlyk, Keren Asraf, Ram Doolman, Efrat Luttwak, Ilya Kirgner, Irit Avivi, Nimrod Adi, Ben-Zion Katz

**Affiliations:** 1 The Intensive Care Unit, Tel Aviv Sourasky Medical Center, Tel Aviv, Israel; 2 The Hematology Institute, Tel Aviv Sourasky Medical Center, 6 Weizman St, Tel Aviv, Israel; 3 Division of anesthesiology, Tel Aviv Sourasky Medical Center, Tel Aviv, Israel; 4 The Division of cardiology, Tel Aviv Sourasky Medical Center, Tel Aviv, Israel; 5 The Central Laboratory, Tel Aviv Sourasky Medical Center, Tel Aviv, Israel; 6 The Dworman Automated-Mega Laboratory, Sheba Medical Center, Tel-Hashomer, Ramat-Gan, Israel; 7 Sackler Faculty of Medicine, Tel Aviv University, Tel Aviv, Israel

**Keywords:** COVID-19, FXIII, FVIII

## Abstract

**Background:**

COVID-19 infection is associated with a hypercoagulable state. Severe COVID-19 patients present with high plasma fibrinogen levels, continuous deposition of fibrin and the presence of microthrombi in their lungs, accompanied by significant fibrinolysis, resulting in high D-dimer levels. Due to the role of FXIII in fibrin crosslinking and clot stabilization, we analyzed its activity levels and dynamics in COVID-19 patients hospitalized in the intensive care unit (ICU).

**Methods:**

FXIII levels were measured in thirty four COVID-19 patients hospitalized in the ICU and in fourteen non-severe COVID-19 patients. FVIII levels were measured for comparison. Laboratory data and clinical variables were recorded.

**Results:**

The average FXIII activity level in 34 ICU hospitalized COVID-19 patients was 69.9±33 %, significantly lower compared to an average of 120±20.9 % FXIII activity in 14 non-severe COVID-19 patients. FXIII activity levels were below the low normal value (< 79 % FXIII activity) in 74 % of the ICU hospitalized COVID-19 patients. In contrast, high FVIII activity was measured among all severe COVID-19 patients. Consecutive measurements, performed in fourteen ICU hospitalized COVID-19 patients, pointed to a significant decrease in FXIII activity from the average of 85.7±28.2 %, (which is in the normal range), to an average of 68.0±20.4 %, below the low normal range, within 6.4±3.4 days of ICU hospitalization. Liver functions did not differentiate between patients with low and normal FXIII activity. No inhibitor to FXIII activity was found in the plasma of severe COVID-19 patients. Levels of FXIII-A antigen correlated with FXIII activity, and were low in severe COVID-19 patients.

**Conclusions:**

Low FXIII activity levels were found in COVID-19 patients hospitalized in the ICU, with gradual decline during their hospitalization. A mechanism of consumption may account for the low FXIII activity in these patients.

## Background

In December 2019, a novel Coronavirus, with person-to-person transmission emerged as a human pathogen [1]. Severe acute respiratory syndrome coronavirus-2 (SARS-CoV-2), causes coronavirus disease 2019 (COVID-19), which has a range of manifestations from asymptomatic to critical illness. SARS-CoV-2 affects mostly the respiratory system, causing viral pneumonitis that may lead to severe acute respiratory distress syndrome (ARDS) [2, 3], however, disease course may result in multi-organ involvement, inducing renal dysfunction, myocardial injury, and hemodynamic instability [4–6].

Early observations revealed a hypercoagulable state in COVID-19 patients, accompanied by elevated D-dimer and fibrinogen levels [3, 7, 8]. These findings correlated with a higher rate of intensive care admissions, as well as mortality [2, 6, 7, 9]. The rate of thrombotic complications appears to be higher amongst COVID-19 patients compared with other critically ill patients. Venous thromboembolic events (VTE) were found in 27 %- 69 % of these severe patients [10, 11]. Of the patients with VTE, deep vein thrombosis (DVT) was reported with an incidence of 23 % [12] (12.7 % in other non COVID-19 ICU patients [13]), and pulmonary embolism (PE) with an unusually high incidence of up to 81 % in some series. [10].

The mechanism underlying COVID-19 coagulopathy has yet to be fully elucidated, in order to obtain better control of this severe manifestation. High levels of FVIII were found in the plasma of COVID-19 patients [14], and vWF levels were higher in the plasma of ICU hospitalized compared with non-severe COVID-19 patients [15]. Reports have linked the hypercoagulable state with a hyperinflammatory state, as it appears that higher fibrinogen level were associated with higher IL-6 and CRP levels [16, 17]. Inflammatory cytokines are established modulators of coagulation and fibrinolysis activation [18]. The concept of immune thrombosis was coined, as cytokines were found to change the normal anticoagulant and profibrinolytic properties of the endothelium to an activated state [19], induce tissue factor (TF) gene expression in endothelial cells and monocytes, fibrinogen synthesis, and platelet production [20]. Autopsy findings indicated diffuse alveolar damage, coupled with microvascular involvement with intra- and extravascular fibrin deposition, and the frequent formation of microthombi in lung arterioles of COVID-19 patients [21]. Lung tissues autopsies of 33/38 (83 %) COVID-19 patients reveled platelet-fibrin thrombi [22]. Hence, fibrin deposition in damaged lung tissues is a prominent pathological aspect of the disease [21–23]. Coagulation factor FXIII plays a central role in the stabilization of the fibrin-based clot, and may also support platelet adhesion at sites of vascular damage [24]. Due to the significant presentations of fibrin deposition and platelets-fibrin microthrombi in the lungs of COVID-19 patients, we studied the levels of FXIII activity in the plasma of 34 severe COVID-19 patients. To the best of our knowledge this is a first report of low levels of FXIII in the plasma of severe COVID-19 patients. Possible mechanisms of FXIII deficiency involvement in COVID-19 morbidity are discussed.

## Materials and methods

### Patients and clinical assessment

This retrospective study was approved by the Tel Aviv Sourasky Medical Center local IRB. Consecutive COVID-19 patients, hospitalized in the ICU at the Tel Aviv Medical Center between March 10th 2020 and April 26th 2020 (7 patients, first outbreak in Israel), and January 5th to February 4th 2021 (27 patients, second outbreak in Israel) were included in the study. All patients were diagnosed with COVID-19, having a positive reverse-transcriptase–polymerase chain reaction assay for SARS-CoV-2 in a respiratory tract sample either prior to, or at admission. All patients were admitted to the ICU due to respiratory failure requiring ventilatory support (mechanical ventilation or high flow oxygen therapy). Demographic and clinical data of participating patients were obtained from the electronic medical records of each subject; these included: Age, gender, comorbid conditions, medications, laboratory findings, daily score of disease severity, invasive and noninvasive mechanical ventilation status, use of extracorporeal membrane oxygenation (ECMO), administration of hemodynamic support, acute cardiac injury, acute kidney injury, major bleeding events, thrombotic events and COVID-19 -related mortality. During their hospitalization, all patients underwent a comprehensive transthoracic echocardiography within 24 h of admission, being part of the routine baseline clinical assessment in ICU. Patients who then experienced clinical deterioration, determined as the need for intubation and mechanical ventilation, deterioration to severe level of hypoxemia (PaO2/FiO2<100), or new onset of circulatory shock, underwent repeated echocardiographic assessment as well as compression ultrasonography of femoral and popliteal veins of both legs.

### Clinical definitions

Severe COVID-19 infection (WHO definition): SatO2<93 on ambient air, RR>30, PF<300.

PE: Lacking a CTA, a diagnosis of PE was based on the combination of acute deterioration in hypoxemia severity without significant change in respiratory resistance or compliance, and the presence of echocardiographic signs suggestive of acute right ventricular strain due to elevated pulmonary vascular resistance (McConell sign, RV dilatation and dysfunction).

### Laboratory assays

3.5 ml of whole blood was collected into vacuum tubes (Greiner, Kremsmunster, Austria) containing 1/10 volume of 3.2 % trisodium citrate. The samples were centrifuged for 8 min at room temperature at 2,500 g, and the plasma was utilized for analysis within two hours. Prothrombin Time assay was performed utilizing Innovin as a recombinant human tissue factor (Dade, Siemens, Marburg, Germany), and measured in seconds. Activated Partial Thrombin Time assay was performed utilizing FSL actin (Dade, Siemens, Marburg, Germany), and measured in seconds. All these assays were performed on either CS2100i or CS5100 instruments (Sysmex, Kobe, Japan). Factor VIII activity was measured by the one-stage clotting assay based on APTT and factor VIII-deficient plasma (Siemens) on a CS2500 instrument (Sysmex). FXIII activity was measured by a FXIII kit (Berichrom, Siemens, Marburg, Germany) on a CS2100i instrument (Sysmex). FXIII-A antigen levels were measured by the commercial automated latex enhanced immunoassay (Werfan, MA, USA) according to the manufacturer’s instructions. Total neutrophils, monocytes and platelet counts were measured by Beckman coulter DxH800 CBC analyzers (Brea, CA, USA). All coagulation factor assays and CBC analyzers in the laboratory are subjected to the ECAT or CAP external quality control program, respectively, with excellent scores. The chemistry blood parameters: wide range CRP, alkaline phosphatase (ALP), Alanine Aminotransferase (ALT), Aspartate Aminotransferase (AST), Gamma-glutamyltransferase (GGT), Albumin and total bilirubin (TBIL) were measured by ADVIA system (Siemens Healthcare Diagnostics Inc., Tarrytown, NY 10591-5097 USA).

### Statistics

Data collected included demographics, past medical history, clinical characteristics, and anticoagulation administered. Categorical variables are shown as frequencies and percentages, and continuous variables as means and standard deviations, or as medians and ranges in the cases of non-normal distributions of parameters. Student`s T-test was utilized to calculate statistical significance in continuous normal distributed variables, Mann Whitney for scale and continuous non-normal distributed variables and Chi square test for nominal variables, *p*<0.05 was considered significant. SPSS software (IBM SPSS Statistics for Windows, version 25, IBM corp., Armonk, NY, USA, 2017) was used for all statistical analyses.

## Results

### Patient characteristics

34 patients, diagnosed with respiratory failure caused by COVID-19 infection and admitted to ICU (defined as severe patients), were included in the study. 14 non-severe COVID-19 patients served as controls. All the severe patients presented with COVID19 pneumonia and required respiratory support in the form of mechanical ventilation or HFOT. The median age of our cohort of patients was 57 years (range, 22-76) and 29 % (*n*=10) were female. None of the patients had a history of respiratory disease, chronic liver disease, inflammatory or immuno-suppressive disease. None of the patients had a prior history of VTE or peripheral vascular disease (Table 1).

**Table 1:** Characteristics, clinical background and medications of severe COVID-19 patients. HTN, Hypertension; DM, Diabetes Mellitus; IHD, Ischemic Heart Disease; CKD. Chronic Kidney Disease; CVA. Cerebro Vascular Accident; TIA, Transient Ischemic Attack; VTE, Venous Thrombo Embolism; Hx, History; NOAC, Novel Anti-Coagulant; ACEi, Angiotensin II converting enzyme inhibitor; ARB, Angiotensin II Receptor Blocker. None of the differences between the groups of normal or low FXIII was statistically significant. Normal FXIII activity was defined as activity level >79 %. Low FXIII levels were levels were defined as activity level <79 %

FXIII activity	Overall (*N*=34)	Normal (>79 %) (*N*=9)	Low (<79 %) (*N*=25)
Age, median (range)	57 (22-76)	54 (42-67)	59 (22-76)
Female gender	10 (29 %)	2 (22 %)	8 (32 %)
HTN	15 (44 %)	6 (67 %)	9 (36 %)
DM	16 (47 %)	6 (67 %)	10 (40 %)
IHD	4 (12 %)	0	4 (16 %)
CKD	3 (9 %)	1 (11 %)	2 (8 %)
Obesity	17 (50 %)	7 (78 %)	10 (40 %)
Hx of CVA/TIA	0	0	0
Hx of VTE	0	0	0
Hx of Malignancy	1(3 %)	0	1 (4 %)
Acetylsalicylic acid	6 (18 %)	1 (11 %)	5 (20 %)
P2Y12 inhibitors	0	0	0
NOACs	0	0	0
ACEi/ARB	5 (15 %)	1 (11 %)	4 (16 %)

### FXIII activity in ICU hospitalized COVID-19 patients

**Fig. 1:**
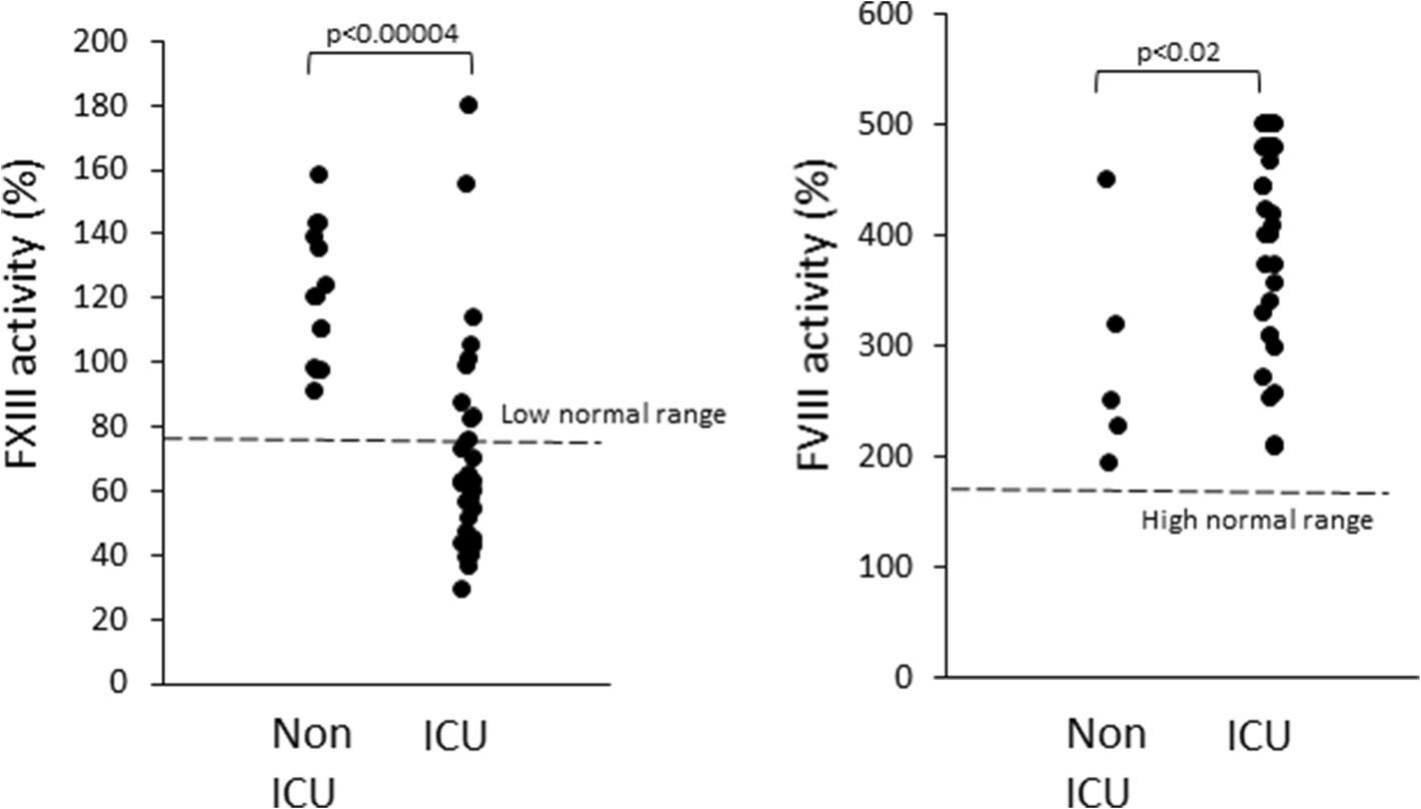
FXIII activity levels of 34 severe ICU hospitalized and 14 non-severe COVID-19 patients (left), and FVIII activity levels of 34 severe ICU hospitalized and 5 non-severe COVID-19 patients (Right). Data are presented in % of activity for both factors. Dashed lines represent the low normal range of FXIII activity (left), and the high normal range of FVIII activity (right). Significant P values are presented

The average FXIII activity level among COVID-19 patients hospitalized in the ICU was 69.9±33 %, median 62.7 %, range 29-180 % with 74 % of the patients (25/34) below the low normal range (79 %) (Fig. 1). The average FVIII activity levels at the same time point were 404.8±95.1 %, median 434 %, range 210-500 % with all patients above the upper normal value (177 %) (Fig. 1). In contrast, in 14 non-severe COVID-19 patients, FXIII activity levels were significantly (*p*<0.0005) higher, with an average of 120.0±20.9 %, median 120 %, range 91-158 %, all within the normal range (Fig. 1). Also, FVIII activity was measured in 5 of these non-severe patients, with an average of 287.8±101.7 %, median 249 %, range 193-450 %, significantly (*p*<0.02) lower compared with the severe COVID-19 patients (Fig. 1). PT values at the time of FXIII measurement were within the normal range (median 11.2; range 10.1-19.8 s; normal range 10.03-12.43 s) in 85 % (29/34) of patients, and the aPTT values at the time of FXIII measurement were also within the normal range (median 28.2; range 20.5-54.3 s; normal range 25-34 s) in 59 % (20/34) of patients (Table 2).

**Table 2:** Laboratory parameters of ICU hospitalized COVID-19 patients. Low FXIII, activity level <79 %; Normal FXIII, activity level >79 %. Median and ranges are presented. Statistical significance was established by Student`s T test (NS, not significance). PT, prothrombin time; PTT, partial thromboplastin time; Neut, total neutrophils count; CRP, C-reactive protein; ALB, albumin, GGT, gamma-glutamyl transferase; ALP, alkaline phosphatase; ALT, alanine transaminase; AST, aspartate aminotransferase; TBIL, total bilirubin. Normal FXIII activity was defined as activity level >79 %. Low FXIII levels were levels were defined as activity level <79 %

Parameter	Normal range	Overall (*N*=34)	Low FXIII (<79 %) (*N*=25)	Normal FXIII (>79 %) (*N*=9)	Significance
PT (sec)	10.03-12.43	11.2 (10.1-19.8)	11.15 (10.1-19.8)	11.3 (10.3-12.9)	NS
PTT (sec)	25-34	28.2 (20.5-54.3)	27.9 (21.4-54.3)	28.7 (20.5-33.4)	NS
Neut X10^3^/µl	1.4-6	8.4 (3.2-27.5)	9.2 (3.2-27.5)	8.3 (5-13.5)	NS
CRP (mg/L)	0.03-5	101.2 (0.3-499.4)	102.33 (1.35-499.4)	81.13 (0.3-293.2)	NS
ALB (mg/dL)	35-50	29.3 (15.2-38.5)	26 (15.2-38.5)	32 (24-34.8)	*P*<0.05
GGT (U/L)	6-42	149.5 (18-1204)	158 (39-1204)	81 (18-567)	NS
ALP (U/L)	46-116	108 (45-412)	111 (49-412)	75 (45-372)	NS
ALT (U/L)	<40	46.5 (45.5-967)	45 (10-967)	46 (14-293)	NS
AST (U/L)	<41	48.5 (16-1344)	46 (18-1344)	57 (16-130)	NS
TBIL (mg/dL)	<1.2	0.36 (0.1-5.24)	0.31 (0.1-5.24)	0.37 (0.24-0.86)	NS

**Fig. 2:**
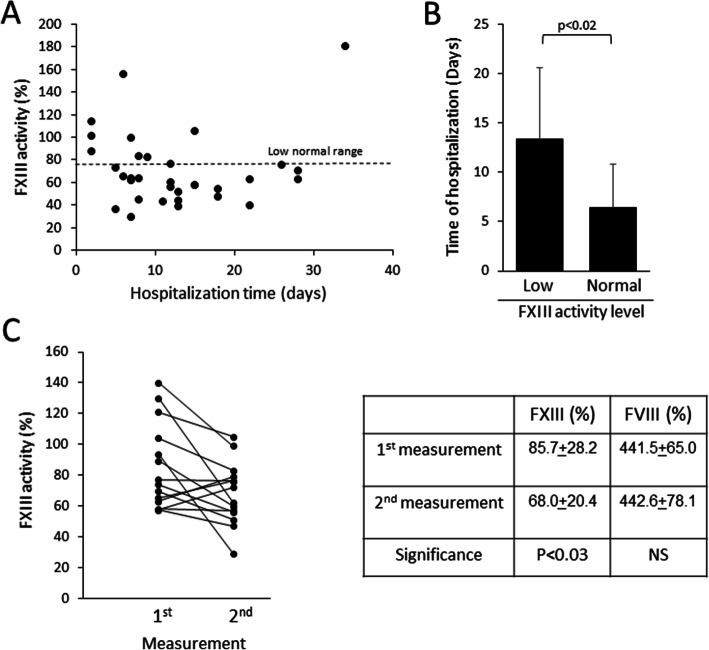
Dynamics of FXIII activity levels in severe COVID-19 patients. **A** FXIII activity levels as a function of time of hospitalization (in days). Dashed line, the lower threshold of normal FXIII activity range. **B** Duration of hospitalization In relation to FXIII measurement in severe COVID-19 patients with normal or with low FXIII levels (*p*<0.02). Normal FXIII activity was defined as activity level >79 %. Low FXIII levels were levels were defined as activity level <79 %. **C** Consecutive measurements of FXIII activity in 14 severe COVID-19 patients. Individual patients dynamics are shown (left), average FXIII and FVIII levels and significance are presented (right). The average interval between the two measurements is 6.1±3.4 days

Levels of FXIII activity varied significantly among the severe patients. As shown in Fig. 2 A, FXIII activity tends to decrease with prolongation of hospitalization. Severe patients with FXIII activity below the normal level had a significantly (*p*<0.02) longer hospitalization period (13.8±7.1 days) than severe patients with levels within the normal range (6.4±4.5 days) (Fig. 2B). Late recovery of FXIII activity levels may occur towards the end of hospitalization, as observed in a single patient at day 34 (Fig. 2 A). Consecutive FXIII activity measurements of 14 ICU hospitalized COVID-19 patients indicated a significant (*p*<0.03) decrease in FXIII activity from an average of 85.7±28.2 % to an average of 68.0±20.4 % within an average of 6.1±3.4 days (Fig. 2 C). No such decrease was observed in FVIII levels (Fig. 2 C).

**Fig. 3:**
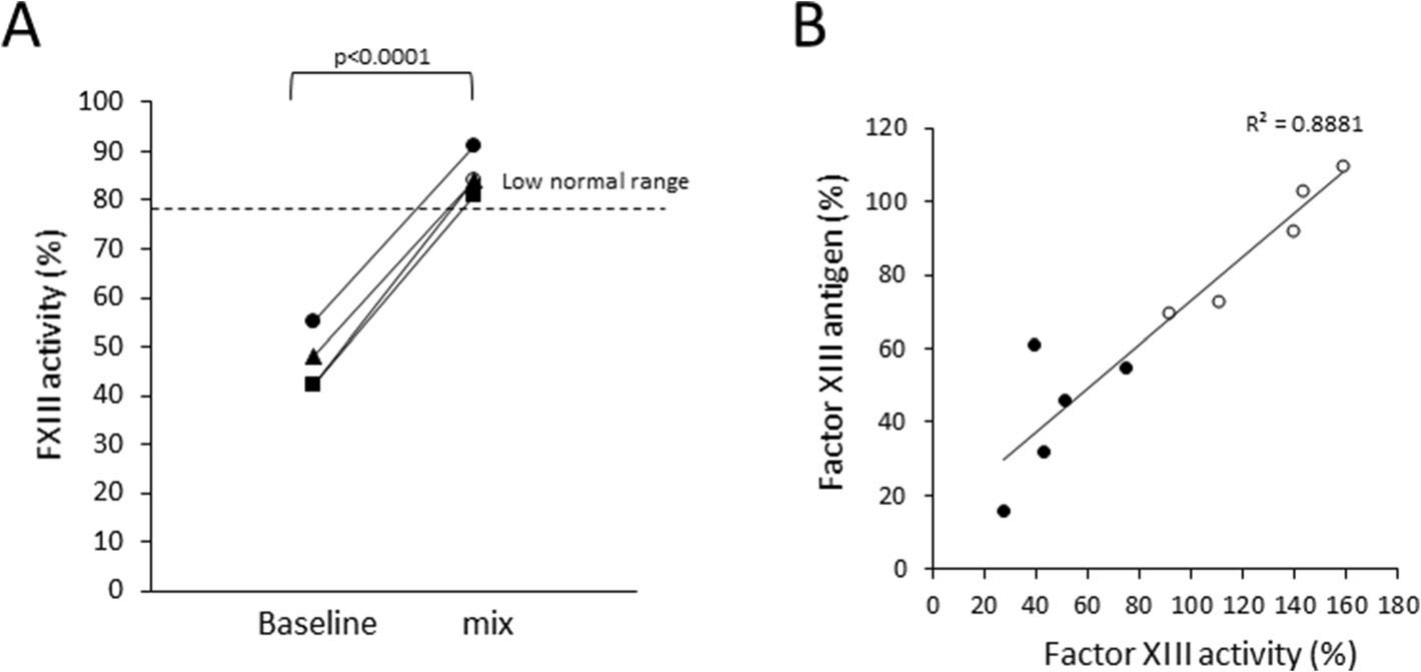
**A** Mixing FXIII activity assay performed on plasma of four severe COVID-19 patients with low FXIII activity levels show significant increase to normal range values. Baseline and mix (1:1 with normal plasma) results are shown (*p*<0.0001). Dashed line, the lower threshold of normal FXIII activity range. **B** FXIII-A antigen levels in COVID-19 patients. FXIII-A antigen levels were determined, and correlated with FXIII activity in the same samples. R^2^ value of the regression comparison between the antigen and activity levels are indicated. Empty circles, non-severe COVID-19 patients; Full circles, severe ICU-hospitalized COVIED-19 patients

FXIII activity in four ICU hospitalized COVID-19 patients in a mixing assay with 50 % normal plasma was significantly increased from the average of 46±6.2 % (all below the normal value) to 85.0±4.2 % (all within the normal range) (Fig. 3 A). Analysis of FXIII-A antigen in the plasma correlated well with FXIII activity measurements (Fig. 3B). The average levels of FXIII-A antigen in severe COVID-19 patients were 42±18 %, significantly lower (*p*<0.004) compared with the levels found in non-severe COVID-19 patients (90±18 %, Fig. 3B).

### Laboratory parameters

No differences were found in CRP levels and total neutrophil counts (inflammation associated parameters) at the time of FXIII activity measurement, between the ICU hospitalized COVID-19 patients with normal FXIII activity, compared with patients with low levels of FXIII activity (Table 2). Abnormal liver function parameters were measured in most of our patients at the time of FXIII activity measurement, with no significant difference between the groups, except for reduced albumin levels in the patients with low FXIII activity (Table 2). In the peripheral blood, FXIII-A is predominantly stored in monocytes and platelets. The average absolute monocyte counts in the severe COVID-19 patients were 640±279.9/µl in the patients with low FXIII levels, and 611.1±279.9/µl in the patients with normal FXIII levels, within the normal range (0-1300/µl), without a significant difference between the groups. The average platelets concentrations in the severe COVID-19 patients were 218.4±108.3 × 10^3^/µl in the patients with low FXIII levels, and 287.2±101.5 × 10^3^/µl in the patients with normal FXIII levels, within the normal range (150-450 × 10^3^/µl), without a significant difference between the groups.

### Clinical parameters

In 6/34 (18 %) ventilated patients, ECMO support was employed, of whom four expressed low FXIII activity levels. Bleeding events were recorded in 8/34 patients (24 %), all with low FXIII activity levels (Table 3). DVT events were recorded in 5/34 (15 %) of our patients, and PE events were recorded in 4/34 (12 %) of our patients (Table 3). Mortality rates were overall 13/34 (38 %); 3/9 (33 %) in the group with normal FXIII activity levels, and 10/24 (40 %) in the group with low FXIII activity (Table 3). No clinical variables reached statistical significance among the groups.

**Table 3:** Morbidity of severe COVID-19 patients. DVT, deep vein thrombosis. PE, pulmonary embolism, VTE, venous thromboembolism, NIV, non-invasive ventilation, ECMO, extracorporeal membrane oxygenation, AKI, acute kidney injury, LOS, length of stay, ICU, intensive care unit. The difference between the groups were not significant. Normal FXIII activity was defined as activity level >79 %. Low FXIII levels were levels were defined as activity level <79 %

FXIII activity	Overall (*N*=34)	Normal (>79 %) (*N*=9)	Low (<79 %) (*N*=25)
DVT	5 (15 %)	2 (22 %)	3 (12 %)
PE	4 (12 %)	3 (33 %)	1 (4 %)
Time from diagnosis to VTE (days)	14.5 (4-23)	17 (4-23)	13 (11-17)
Time from admission to VTE (days)	11 (0-24)	16 (12-24)	7 (0-13)
Major Bleed	8 (23.5 %)	0	8 (32 %)
NIV	23 (68 %)	8 (89 %)	15 (60 %)
ECMO	6 (18 %)	2 (22 %)	4 (16 %)
Medical Hemodynamic support	24 (71 %)	6 (67 %)	18 (72 %)
AKI	14 (41 %)	2 (22 %)	12 (48 %)
Mortality during follow up period	13 (38 %)	3 (33 %)	10 (40 %)
Follow up duration (days)	18 (5-45)	13 (5-35)	18 (8-45)
LOS ICU (days)	25 (9-45)	35.5 (35-36)	18 (9-45)

## Discussion

The high D-dimer levels and possibly systemic procoagulant state of COVID-19 patients prompted the postulation of a novel pulmonary intravascular coagulopathy [25]. This theory is supported by autopsies performed on the lungs of COVID-19 patients, demonstrating small, firm thrombi and fibrin deposition in sections of the peripheral parenchyma in some of the cases [21–23]. As previously shown, aberrant fibrinolysis may occur locally in tissues affected by the disease [26], with no effects on global fibrinolysis. In spite of ongoing consumption, the levels of plasma fibrinogen are above the normal range in most of these patients. This may stem from parallel persistent fibrinogen production, driven by the substantial inflammation typical to severe COVID-19 [27]. Due to the coagulopathy in COVID-19 patients, comprehensive studies mapped various coagulation functions in these patients. For example, a recent study provided a comprehensive outlook of the coagulation system in 206 COVID-19 patients, but the levels of FXIII and its possible role in COVID-19 have not yet been examined [28].

We found low FXIII activity in most of our ICU hospitalized COVID-19 patients, but not in non-severe COVID-19 patients. Extensive serum (not plasma) proteomic analysis of COVID-19 patients pointed to the possibility of decrease in FXIII-B in the sera of COVID-19 patients, in a correlation to increase in IL-6 levels [29]. In our study, we did not find a correlation between low FXIII activity and inflammation associated parameters (CRP, total neutrophils count). Three possible mechanisms may account for the low levels of FXIII activity in such patients: (1) Inhibition, (2) Reduced production (mostly due to hepatic failure), 3.Consumption. The first mechanism may stem from inhibitory antibodies against FXIII, as autoantibodies are a common finding in COVID-19 patients [30], and the emergence of autoantibodies is a well-established mechanism in acquired FXIII deficiency [31]. While the targets of such antibodies in COVID-19 are diverse and include components of the immune system, some may be associated with coagulopathy, such as anti-phospholipid autoantibodies [30]. However, this mechanism is not supported by our mixing test that complemented the levels of FXIII activity to a normal level, with no evidence of inhibitors in the plasma of the patients. Hepatic dysfunction has been reported in 14-53 % of COVID-19 patients [32], and since FXIII-B is produced in the liver we examined liver functions in our severe COVID-19 patients. We recorded abnormal liver functions in most of our ICU hospitalized COVID-19 patients, but with no significant difference between patients with low or normal levels of FXIII activity. We found lower albumin levels in patients with low FXIII activity, in agreement with previous studies that demonstrated hypoalbuminemia in severe COVID-19 patients [33]. It was suggested that the mechanism underlying hypoalbuminemia in COVID-19 patients is albumin excretion into damaged organs [33]. Since the major cellular storage of FXIII-A in the circulation is within monocytes and platelets [34], we examined their concentrations in our severe COVID-19 patients, and did not find any significant differences between patients with low or normal FXIII activity levels. Hence, our data indicate that production difficulties may not account for the low FXIII we observed in severe COVID-19 patients, but rather a consumption-based mechanism. This approach is supported by the concomitant reduction in FXIII-A (the catalytic subunit) antigen with the decreased FXIII activity in the severe patients. Consumption accompanies conditions such as inflammation and activation of the coagulation system, both relevant to COVID-19. We show herein that the levels of FXIII activity in the severe COVID-19 patients, are not stable, but rather decline with prolongation of hospitalization, and may be associated with continuous consumption due to the ongoing fibrin deposition in the lungs of these patients.

More data is needed to establish whether low levels of FXIII are involved in mechanisms underlying coagulopathy in severe COVID-19 patients, but previously published experimental data may point to such possibilities; Increased embolism was found in FXIII-/- compared with wild type mice in acute DVT model, apparently due to the effects of FXIII deficiency on clot stability [35]. Moreover, it was shown that FXIII supplementation stabilizes venous thrombi, and reduces embolism [36]. In humans, a weak protective effect of increased FXIII activity against VTE was demonstrated [37]. Moreover, the combination of high D-dimer and low FXIII concentrations was found to be associated with PE, and a threshold of 69 % of FXIII concentration was set to distinguish between confirmed PE and other serious diseases with similar symptoms [38]. In our cohort, the average level of FXIII activity in the severe patients was similar, 69.8 %, combined with high D-dimer levels in most of our patients. Hence, low FXIII levels may alter thrombi properties, resulting in an increased risk for embolism. The predominant manifestation of FXIII deficiency is bleeding [39]. We recorded bleeding events in 8/34 (24 %) of our patients which all occurred in patients with low FXIII activity (10/25, 32 % of these patients). Despite considerable coagulopathy in COVID-19, a high prevalence of bleeding has not been reported in this disease [40]. The overall hypercoagulable state in COVID-19 may overlay bleeding risk factors discussed before [40], or the low levels of FXIII activity presented in this study.

## Conclusions

Low FXIII activity levels were found in most of our ICU hospitalized COVID-19 patients. In contrast, the levels in most of non-severe COVID-19 patients examined in this study were within normal ranges. The levels of FXIII activity in the severe COVID-19 patients decline with prolongation of hospitalization. Our data indicate that low production or circulating inhibitors may not account for the low FXIII we observed in severe COVID-19 patients, but rather a consumption-based mechanism. FXIII deficiency may be involved in COVID-19 morbidity.

## Data Availability

All supporting data is available from the corresponding author upon request.

## Abbreviations

ALP: alkaline phosphatase
ALT: alanine aminotransferase
aPTT: activated partial thromboplastin time
AST: aspartate aminotransferase
ARDS: acute respiratory distress syndrome
CBC: complete blood count
COVID-19: coronavirus disease 2019
CRP: C reactive protein
CTA: computed tomography angiography
DVT: deep vein thrombosis
ECMO: extracorporeal membrane oxygenation
FVIII: coagulation factor 8
FXIII: coagulation factor 13
GGT: Gamma-glutamyltransferase
HFOT: high flow oxygen therapy
ICU: intensive care unit
IL-6: interleukin 6
PE: pulmonary embolism
PT: prothrombin time
SARS-CoV-2: Severe acute respiratory syndrome coronavirus-2
TBIL: total bilirubin
TF: tissue factor
VTE: venous thromboembolic events
vWF: von Wilebrand factor
